# Transcriptome wide identification and characterization of Starch Synthase enzyme in finger millet

**DOI:** 10.6026/97320630014393

**Published:** 2018-07-31

**Authors:** Rajhans Tyagi, Apoorv Tiwari, Alok Kumar Gupta, Sanjay Gupta

**Affiliations:** 1 Uttarakhand Technical University, Dehradun, Uttarakhand, 248007, India; 2 Sam Higginbottom University of Agriculture, Technology and Sciences (SHUATS), Allahabad, 211007, India; 3 Dept of Molecular Biology and Genetic Engineering, G.B. Pant University of Agriculture and Technology, Pantnagar, Uttarakhand, 263145, India; 4 Amity University Uttar Pradesh, Noida. 201313, India; 5 Himalayan School of Biosciences, Swami Rama Himalayan University, Jolly Grant, Dehradun, 248016, India

**Keywords:** Finger millet, CDS, SBE, Domain, PMDB, NCBI

## Abstract

Finger millet is a calcium-rich cereal crop of the grass family. The transcriptome data for finger millet is available at NCBI. It is of interest to annotate and characterize starch synthase enzyme from finger millet transcriptome data. Starch synthase plays an important role in the elongation of glucan chains during the formation of starch. The starch synthase enzyme is characterized using three domains (Glyco_transf_5, Glycos_transf_1 and Glyco_trans_1_4). Binding sites for GLC (alpha-d-glucose), PLP (Pyridoxal-5'- phosphate), AMP (Adenosine monophosphate) and GOL (Glycerol) are found. The phylogenetic analysis showed that the finger millet starch synthase is similar to the granule-bound starch synthase of Oryza sativa and Concrete amaricanus. We report the sequence (GenBank accession number KY648917) and the structural model of finger millet starch synthase (PMDB ID: PM0081600).

## Background

Starch is the important molecule for plant development and reproduction. Starch can define as a complex branched glucose polymer and the branch molecular weight distribution power up the nutritionally important properties such as digestion rate, etc [1]. Resistant starch is considered the third type of dietary fiber, as it can deliver some of the benefits of insoluble fiber and some of the benefits of soluble fiber. Some carbohydrates such as sugar and most starch are rapidly digested and absorbed as glucose into the body through the small intestine and subsequently used for short-term energy needs for stored. Resistant starch, on the other hand, resists digestion and passes through the large intestine where it acts like dietary fibers. The resistance from digestion is attributed by the ratio of amylase and amylopectin chain during starch biosynthesis besides architectural effects during packaging of grains. Starch is the end product of photosynthesis in source tissues and is stored as energy reserves in sink tissues. Starch has two major components, the basically linear a-polyglucan amylose, and the branched a-polyglucan amylopectin. The a-1, 4-glucosidic link chains of both amylose and amylopectin are elongated by the addition of the glucose moiety from ADP glucose, which is synthesized by ADP glucose pyrophosphorylase (AGPase) from glucose-1-P, to the nonreducing end of the a-glucan acceptor molecule. The elongation reactions for the a-1, 4-chains of amylose and amylopectin are distinctively catalyzed using a starch granule-bound form of starch synthase (GBSS) and a soluble form of starch synthase (SS), respectively. Starch synthase activity was first discovered using Leloir's group and the activity were associated with the starch granule. These starch synthases are designated as GBSSs to distinguish them from the starch synthases mainly in the soluble phase of the chloroplast or amyloplast. The starch-synthase enzyme is one of the four major enzyme classes involved in starch biosynthesis in plants as SSI, SSII, SSIII, and SSIV.

A cereal is a grass, a member of the monocot family Poaceae also known as Gramineae, which usually have long, thin stalks, such as wheat, rice, maize, sorghum, millet, barley, and rye, whose starchy grains are used as food. The term cereal is not limited to these grains. But, refers to foodstuff prepared from the starchy grains of cereal like flours, breads and pasta [2]. All cereals are annual plants and consequently, one planting yields one harvest. The demands on climate, however, are different. Warm-season cereals (corn, rice, sorghum, millet) are grown in tropical lowlands throughout the year and in temperate climates during the frost-free season. Finger millet is considered to be a boon for diabetes patients and obese people, as the digestion of finger Millet takes place at a slow pace and hence, glucose is released slowly into the blood [3]. In rice and other related plants the sequence of starch synthase enzyme is known but in finger millet, the nucleotide sequence of the starch synthase enzyme is not yet known. Availability of the transcriptome data of developing spikes of finger millet [4] provides an opportunity to predict the gene sequence of important traits of the nutritional point of view. Hence an effort is made for the prediction of an important enzyme i.e. starch synthase enzyme in the finger millet and submit the genomic as well as proteomic information for further scientific and research interventions.

## Methodology

### Sequence retrieval and Domain prediction

The sequence of finger millet starch synthase enzyme was retrieved by the blast analysis of the homologous sequence of rice download from NCBI database. Local BLAST [5] was used to retrieve starch synthase sequence of finger millet from the online submitted [4] transcriptome data of finger millets. ORF Prediction tool was used for open reading frame prediction with default parameters and verify predicted protein using newly developed SMART BLAST or regular BLASTP. ORF finder searches for open reading frames (ORFs) in the nucleotide sequence (KY648917). The program returns the range of each ORF, along with its protein translation. SMART domain prediction tool was used for the prediction of the domain in the coding sequence of starch synthase finger millet.

### Protein Primary, Secondary and Tertiary Structure and Function Prediction

Protein sequence level annotation was done using ProtParam server. It allows the computation of various physical and chemical parameters for protein sequence (ARA71548). The computed parameters include the molecular weight, theoretical pI, amino acid composition, atomic composition, instability index, aliphatic index and grand average of hydropath city (GRAVY) [6].

The secondary structure of protein sequence (ARA71548) was predicted by CFSSP (Chou & Fasman Secondary Structure Prediction Server) is an online protein secondary structure prediction server. This server predicts regions of secondary structure from the protein sequence such as alpha helix, beta sheet, and turns from the amino acid sequence. The output of the predicted secondary structure is also displayed in linear sequential graphical view based on the probability of occurrence of alpha helix, beta sheet, and turns with implemented CFSSP is Chou-Fasman algorithm, which is based on analyses of the relative frequencies of each amino acid in alpha helices, beta sheets, and turns based on known protein structures solved with X-ray crystallography [7].

The tertiary structure of the starch synthase enzyme (ARA71548) was predicted using RaptorX a web portal for protein structure and function prediction. The amino acid sequence of the enzyme was submitted to the server and which predicts its tertiary structures as well as contact map, solvent accessibility, disordered regions and binding sites and assigned confidence scores to indicate the quality of prediction results. For structure validation purpose Ramachandran plot analysis was done using RamPage server [8].

### Template validation and Phylogenetic Analysis

For validating the template-based modeling by RaptorX server, we have done Blastp against PDB database and select the template protein sequences. These Protein sequences were subjected to multiple sequence alignment and tree construction using MEGA v6.0 [9]. We construct another phylogenetic tree of starch synthase nucleotide sequence on the basis of protein sequence similarity by using NCBI blastp non-redundant protein database and retrieved all the similar sequences for phylogenetic analysis.

## Result & Discussion

### ORD and Domain Prediction

Nucleotide as well as protein sequences of starch synthase of Eleusine coracana were retrieved by NCBI database, which were submitted in NCBI with nucleotide sequence accession KY648917 length is 1851 base pare and protein sequence ARA71548 is comprises 616 amino acids. The open reading frame analysis of the nucleotide sequence resulted in 20 ORF, on positive and negative strands and selected the longest ORF among them with 616 amino acid long and cover a total length of the Nucleotide sequence.

SMART Domain prediction tool predict the three major domains in the protein sequence of starch synthase enzyme viz- low complexity domain (51-75), Glyco_transf_5 (90-351) with and Glycos_transf_1 (396-531) as shown in Table 1. This family is most closely related to the GT1 family of glycosyltransferases. Glycogen synthase catalyzes the formation and elongation of the alpha-1,4-glucose backbone using ADP-glucose, the second and key step of glycogen biosynthesis. This family includes starch synthases of plants, such as DULL1 in Zea mays and glycogen synthases of various organisms.

Superfamily glycosyltransferases catalyze the transfer of sugar moieties from activated donor molecules to specific acceptor molecules, forming glycosidic bonds. The acceptor molecule can be a protein, a heterocyclic compound, a lipid or another carbohydrate residue. The structures of the formed glycoconjugates are extremely diverse, involved in a wide range of biological functions. The members of this family share a common GTB topology, one of the two protein topologies observed for nucleotide-sugar-dependent glycosyltransferases. GTB proteins have distinct N and C- terminal domains each containing a typical Rossmann fold. The two domains have high structural homology despite minimal sequence homology. The large cleft that separates the two domains includes the catalytic center and permits a high degree of flexibility.

### Protein Primary, Secondary and Tertiary Structure Prediction

A number of amino acids in the starch synthase are 616 Table 2 and the molecular weight was found 67201.01 with theoretical pI 6.87. The total number of negatively charged residues (Asp + Glu) and the total number of positively charged residues (Arg + Lys) were 72 and 71 respectively. The chemical formula of the enzyme is "C2987H4700N830O875S30" with 9422 number of atoms. The aliphatic index was found 81.59 and grand average of hydropathicity (GRAVY) was -0.200. The instability index (II) is computed to be 27.50 which classify the protein as stable. An intermediate but useful step is to predict the protein secondary structure, that is, each residue of a protein sequence is assigned a conformational state, either helix (H), sheet (E) or coil (C) and the secondary structure of starch synthase has 71.3% helix, 60.2% sheet and 11.9 % coil region.

**Figure 1:**
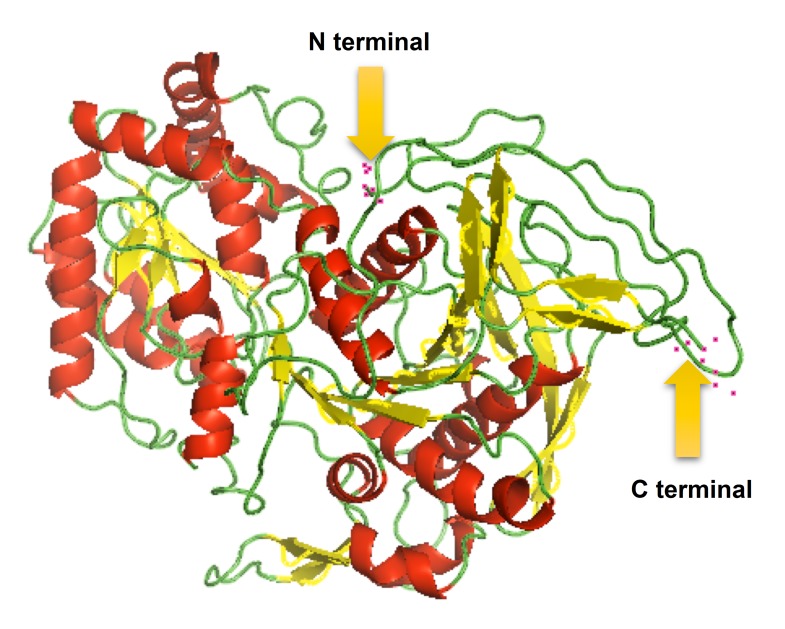
Tertiary structure of Starch Synthase enzyme predicted by RaptorX server using PDB 3VUE A chain as a template

Tertiary structure of starch synthase was predicted by using 3vueA template with p-value 9.74e-12 and Overall uGDT (GDT) is 448 (72) and 616(100%) residues are modeled. Solvent access is 22%E, 44%M, 32%B Figure 1. The Ramachandran plot analysis of the predicted structure shows that the percentage of residues in favoured region is 95.0%, residues in allowed region 4.2% and residues in outlier region was 0.8%, which indicate that the structure has a stable and accurate prediction. The binding affinity of ligands i.e. Alpha-d-glucose, Pyridoxal-5'-phosphate Adenosine monophosphate and Glycerol with the starch synthase tertiary structure was calculated by RaptorX server Table 3 & Table 4. The predicted model of starch synthase of finger millet in the 3D conformation was submitted to protein model submission database with the PMDB ID - PM0081600 [10].

### Phylogenetic analysis

**Figure 2:**
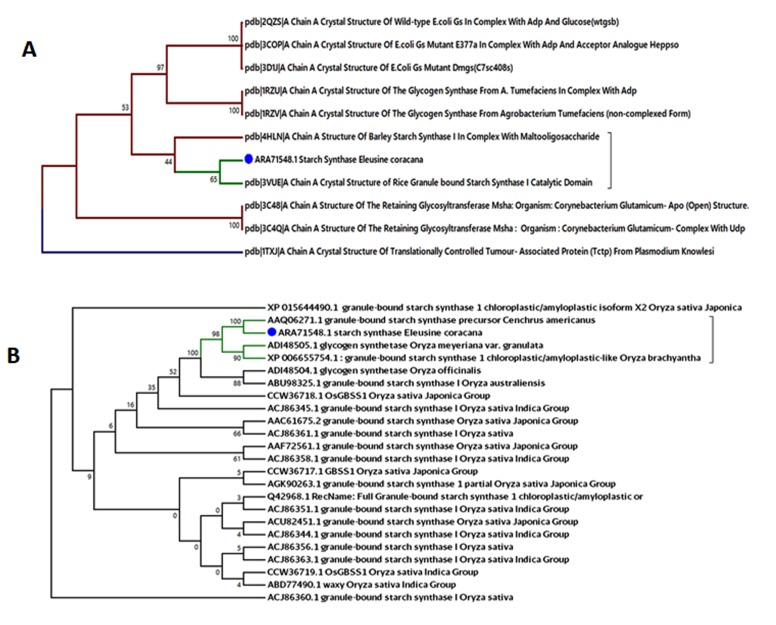
A- Phylogenetic tree for template validation (3VUE A chain) on the basis of structural similarity for structure prediction of Starch synthase enzyme and B- On the basis of protein sequence homology with Starch synthase enzyme of Eleucine coracana.

For prediction of protein tertiary structure, there is need of template so Blastp program was used against PDB database and use all the hits for the phylogenetic study and 3VUE of rice was found much closer with starch synthase of Eleusine coracana as shown in Figure 2A.

Phylogenetic analysis was done on the basis of protein sequence and protein structure. The sequence level analysis was done on the basis of blastp result and top 25-blast hit were retrieved and used these sequences for phylogenetic analysis by aligning them together. By this analysis, it was found that the starch synthase sequence of Eleusine coracana was much similar to the granulebound starch synthase of Oryza sativa and Concrete amaricanus as shown in Figure 2B.

## Conclusion

Finger millet is a nutritionally rich cereal crop grown in different regions of worlds under adverse weather conditions. It is rich in calcium among other cereal crops. The transcriptome data of finger millet is also available. Different pathways are involved in the starch biosynthesis catalyzed by different enzymes. Starch is synthesized in plastids, including chloroplasts in photosynthetic tissues and amyloplasts in non-photosynthetic tissues such as seeds, roots, and tubers. Starch synthesized in chloroplasts of photosynthetic tissues is degraded to hexoses during the dark period. It is of interest to characterize starch synthase enzyme from finger millet. We report its sequence and structural model with predicted functional and architectural features.

## Figures and Tables

**Table 1:** Domain analysis of finger millet starch synthase enzyme

Name	Start	End	E-value
low complexity	51	75	N/A
Pfam:Glyco_transf_5	90	351	5.60E-73
Pfam:Glycos_transf_1	396	531	1.10E-15
Pfam:Glyco_trans_1_4	405	535	2.50E-06

**Table 2:** Amino acid composition of starch synthase protein sequence

Amino Acid	Number	Percentage
Ala (A)	69	11.20%
Arg (R)	36	5.80%
Asn (N)	22	3.60%
Asp (D)	33	5.40%
Cys (C)	12	1.90%
Gln (Q)	17	2.80%
Glu (E)	39	6.30%
Gly (G)	56	9.10%
His (H)	12	1.90%
Ile (I)	26	4.20%
Leu (L)	48	7.80%
Lys (K)	35	5.70%
Met (M)	18	2.90%
Phe (F)	22	3.60%
Pro (P)	38	6.20%
Ser (S)	26	4.20%

**Table 3:** Binding affinity of starch synthase and their ligands

Pocket	Multiplicity	Ligand	Binding residues
1.	81	GLC	G106 L107 V110 H270 N271 V327 N359 Q418 E492 P493 C494 G495
2.	69	PLP	A99 K103 G105 G106 G413 K419 F470 N471 A472 G495 L496 I497 Q500
3.	36	AMP	H160 I217 L218 N219 L220 N221 S222
4.	17	GOL	G105 G106 L107 D240 W241 H242 N271 Y274 G276

**Table 4:** Chemical Component Summary of ligands binds with the starch synthase

S. No.	Ligands	Name	Formula	Molecular Weight	Type
1.	GLC	Alpha-d-glucose	C6 H12 O6	180.16	SACCHARIDE
2.	PLP	Pyridoxal-5'-phosphate	C8 H10 N O6 P	247.14	NON-POLYMER
3	AMP	Adenosine monophosphate	C10 H14 N5 O7 P	347.22	NON-POLYMER
4	GOL	Glycerol	C3 H8 O3	92.09	NON-POLYMER
